# Cognitive disorders in childhood epilepsy: a comparative longitudinal study using administrative healthcare data

**DOI:** 10.1007/s00415-022-11008-y

**Published:** 2022-02-15

**Authors:** Anna-Lisa Sorg, Rüdiger von Kries, Ingo Borggraefe

**Affiliations:** 1 Institute of Social Pediatrics and Adolescent Medicine, Ludwig-Maximilians-University Munich, Munich, Germany; 2 Division of Pediatric Neurology, Developmental Medicine and Social Pediatrics, Department of Pediatrics and Interdisciplinary Epilepsy Center, Ludwig-Maximilians-University Munich, Lindwurmstreet 4, 80337 Munich, Germany

**Keywords:** Children, Epilepsy, Cognition

## Abstract

**Objective:**

The study aimed to assess the risk of cognitive impairment in patients with epilepsy, the impact of age of epilepsy onset on cognition and the temporal relationship of epilepsy onset and intellectual impairment.

**Methods:**

This longitudinal study analyzed birth cohorts and followed-up children born 2005–2007 up to the age of ten using administrative healthcare data of about 8.9 million members insured by the statutory health insurance “BARMER” in Germany. We compared prevalence of cognitive impairment (ICD-code F7*) in children with epilepsy (ICD-code G40) to controls, and calculated relative risks by age groups at onset of epilepsy and assessed differences in relation to the temporal sequence of the diagnoses.

**Results:**

Of the 142,563 pre-pubertal children included in the analysis, 2728 (1.9%) had an epilepsy diagnosis within the first 10 years of life. 17.4% (475/2728) of children with epilepsy had a diagnosis of cognitive impairment compared to 1.7% (2309/139835) in controls. The relative risk for cognitive impairment compared to age-matched controls was 10.5 (95% CI 9.6, 11.6) and was highest in epilepsy cases with seizure manifestation within the first 2 years of life compared to older children. The prevalence of cognitive impairment before epilepsy diagnosis was slightly increased compared to controls, while it was increased by a factor of nine in children diagnosed with cognitive impairment in the year of onset of epilepsy or afterwards.

**Conclusions:**

Pre-pubertal children with epilepsy have a ten-fold higher risk for intellectual impairment compared to age-matched controls. This risk inversely correlates with the age of epilepsy manifestation. Cognitive impairment was diagnosed after epilepsy manifestation in the majority of patients.

## Background

Mental health, developmental and behavioural problems are comorbidities present in some children with epilepsy [1]. These comorbidities might have a higher impact on impaired quality of life for both, patients and caregivers than seizure activity itself [2, 3]. Additionally, adolescents with epilepsy often feel more socially disadvantaged than patients with other, non-neurological chronic diseases [4]. Patients with childhood-onset epilepsies with cognitive problems have a higher rate of unemployment than childhood-onset epilepsy patients without cognitive problems in adulthood [5].

The causes of these epilepsy associated comorbidities are often difficult to dissect as many factors might contribute to impaired mental function [6]. First, epilepsy-related factors as age of seizure onset, seizure frequency and duration might have a significant impact on comorbidities suggesting higher prevalence in patients with earlier seizure onset, higher seizure frequency and longer epilepsy duration. Second, the cause of the underlying epilepsy syndrome impacts to cognitive performance: patients with idiopathic epilepsy syndromes show higher intelligence quotient (IQ) scores than patients with monogenetic epilepsies (i.e. caused by mutations in ion channels encoded by *SCN1A*) [7]. Some researchers even propose to call the latter epilepsy group ‘Developmental brain disorders with the comorbidity of epilepsy’ in order to pronounce the high impact of cognitive, developmental and behavioral disturbances in theses syndromes beside the burden of seizures [8]. Lastly, significant effects on cognitive performance and behavioral disturbances have been shown for many antiseizure medication (ASM) and commonly reveal cumulative effects when drugs are combined [9, 10].

There is plenty of data about cognitive impairment in children with epilepsy [6, 11, 12]. Most of these data, however, lack a control group of children without epilepsy and even fewer studies focus the pre-pubertal age. Follow-up of birth cohorts in healthcare data allows assessing the absolute risk for cognitive impairment in children with epilepsy and the relative risk compared to children without epilepsy. The longitudinal structure of these data facilitate addressing the assessment of the temporal relation of epilepsy and cognitive impairment.

The study aimed to assess the following questions: how much does epilepsy increase the risk for cognitive impairment for children of pre-pubertal age? Does this risk increment vary by age of onset of epilepsy? Is there a risk that cognitive impairment precedes epilepsy onset?

## Methods

### Data source

In Germany, there is obligatory statutory health insurance, which covers 90% of the population [13]. Persons with high income are exempt and may opt between none, private or statutory health insurance [14]. There are over 100 different statutory health insurance providers [15]. One of these is BARMER. In terms of the number of insured persons, BARMER is the second largest statutory health insurance in Germany. About 11% of the German population (8.9 million health insurance holders) is BARMER insured [16, 17]. BARMER insured slightly more children aged younger than 15 years than other statutory health insurances on average (13.74% vs 12.77%) [18].

Physicians or clinics claim the treatment fees from the health insurance provider and therefore collect several data, including the ICD codes (International Classification of Diseases) for the diagnoses related to the medical treatment and drug prescriptions. All medical diagnosis are coded in the German Modification of ICD-10 published by the World Health Organization [19]. The insurance thus collects information on physician contacts and diagnoses for each insured person. BARMER provides this data in pseudonymized form for scientific purposes. Data of the present study comprise insured persons from 2005 to 2019 and could, therefore, follow-up birth cohorts in a retrospective cohort study.

### Study cohort

This analysis is based on data for children born in 2005–2007 with complete follow-up (= permanently BARMER insured until 2019 or until death). Patients with a diagnosis of epilepsy in the first 10 years of life defined the cases. Patients without a diagnosis of epilepsy in the first 10 years of life served as the control group. Outpatient and inpatient physician visits for epilepsy from birth through the age of ten (means until 2015 for the 2005 birth cohort, until 2016 for the 2006 birth cohort and 2017 for the 2007 birth cohort) and for case validation 2 years beyond were considered.

### Case definition

We defined ‘pre-pubertal’ as from birth up to 10 years of age. The ICD code for epilepsy is G40*. All children not meeting the case definition were controls. The case definition used in our analysis was adapted from the recommendations by Reid et al. [20]. We proposed ‘2 physician claims or 1 hospitalization in 2 years coded’ (ICD G40) as the most accurate and ‘1 physician claim or 1 hospitalization or 1 ER visit in 2 years’ as the most sensitive algorithm. In contrast to the suggestion by Reid, we decided not to include G41 (status epilepticus) cases. R56.1 (febrile convulsions) was also not considered within the “epilepsy” group unless the concomitant code of G40 was documented as defined above.

Outpatient and inpatient physician visits for epilepsy from birth through the age of 10 (means until 2015 for the 2005 birth cohort, until 2016 for the 2006 birth cohort and 2017 for the 2007 birth cohort) and for case validation 2 years beyond, respectively, were considered. A confirmed diagnosis of epilepsy requires a diagnose of G40 anytime in the first 10 years of life ‘in at least two outpatient physician claims’ or ‘related to one hospitalization and at least one outpatient physician claim’ or ‘related to more than one hospitalization’ in the year of the first G40 diagnosis and two subsequent years.

### Main outcomes

Beyond description of the pre-pubertal incidence rate of epilepsy, the study aimed to investigate the association between epilepsy and cognitive impairment. Cognitive impairment was defined by a diagnosis of ICD-code F7*. Cases with different F7* codes during the first 10 years of life or with code F78 or F79 only were summarized and labeled as “unspecific cognitive impairment”.

### Statistical methods

The year of onset of epilepsy was defined as the year with the first G40 diagnosis.

Confidence intervals (CI) of 95% for incidence were calculated according to Agresti Coull [21, 22].

The prevalence of cognitive impairment in epilepsy cases and controls is displayed with 95% CI. Relative risk (RR) were calculated to compare case and control group. Differences between cases and controls were tested using chi-square statistics. The significance level for all analyses was set at 5%. We performed all analyses with SAS Enterprise guide 8.3 Update 2.

### Data accessibility and ethics

This is an analysis on healthcare data, not owned by the authors. BARMER gives remote access to their data to scientists with an appropriate research question.

Informed consent is not necessary in retrospective data analysis of pseudonymized healthcare data. The ethics committee of the medical faculty of the Ludwig-Maximilians-University Munich gave ethical approval (Number 17-742 UE until 17-746 UE).

## Results

### Incidence of epilepsy in pre-pubertal children

**Fig. 1:**
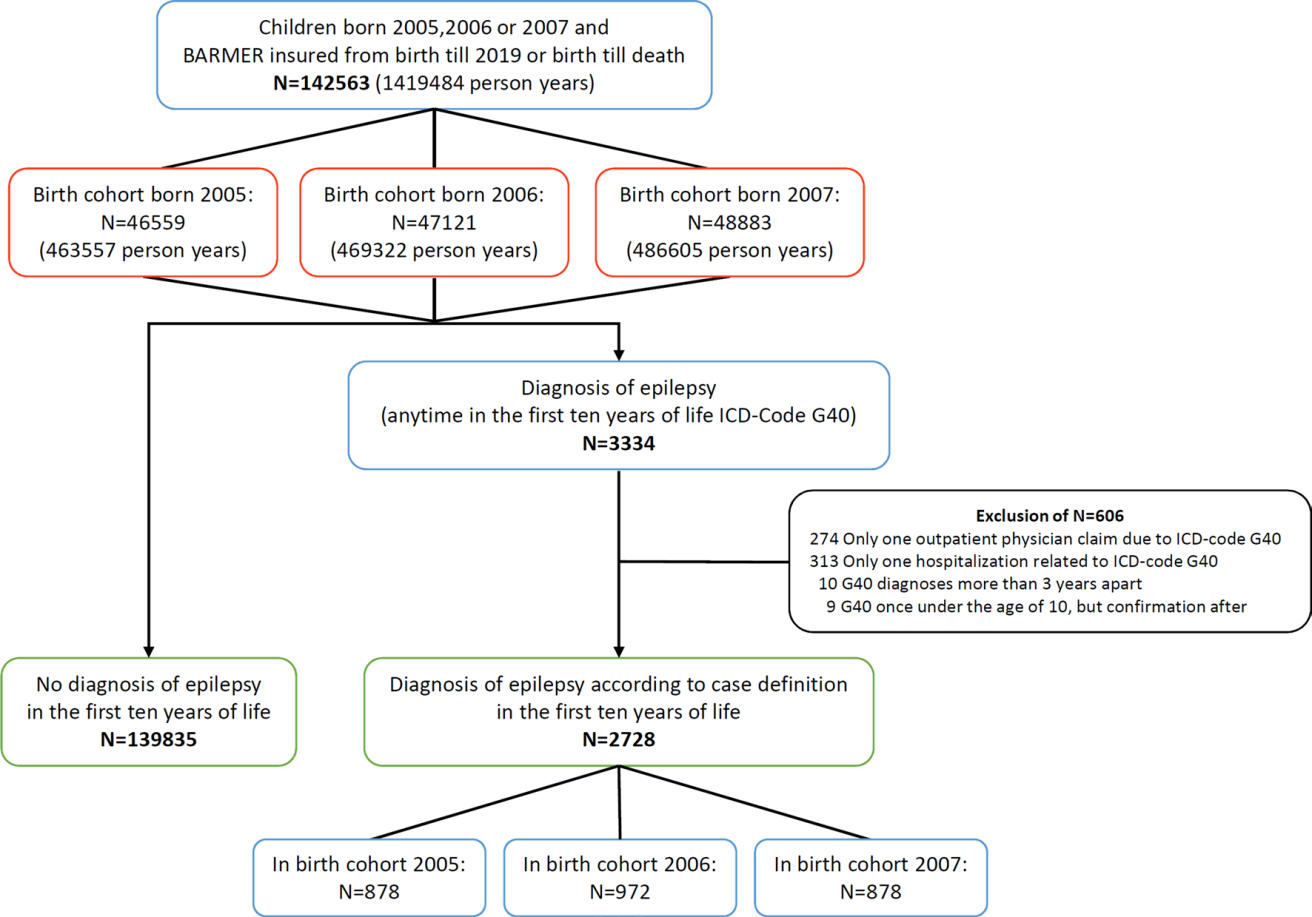
Flowchart showing the study population of children up to the age of ten with/without epilepsy. A total of 142,563 individuals were available for primary analysis

**Fig. 2:**
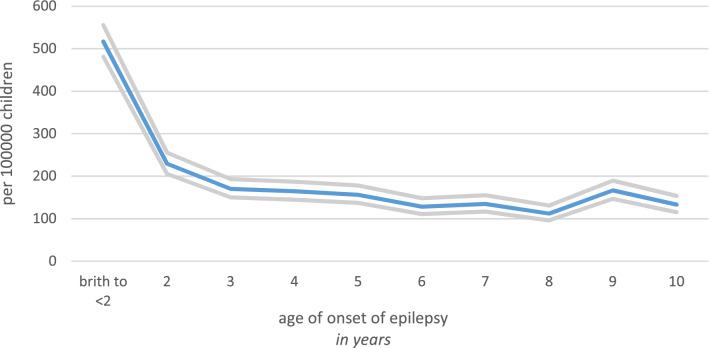
The incidence rate of pre-pubertal epilepsy. The incidence rate of pre-pubertal epilepsy is highlighted in blue, the upper and lower confidence intervals are depicted in gray. The highest incidence rate was reported at the age of 1 year

We identified 142,563 children born in 2005–2007 with complete follow-up until 2019 or until death. 2728 (1.9%) had an epilepsy diagnosis within the first 10 years of life (Fig. 1). The incidence rate of pre-pubertal epilepsy was 191 [95% CI 185, 200] per 100,000 person years and the highest age-specific incidence of 314 [95% CI 286, 344] per 100,000 children was recorded at the age of one. The age-specific incidence subsequently decreased to 112 (95% CI 96, 131] per 100,000 by the age of eight (Table 1 and Fig. 2). There were slightly more male children in the case group (54%, *n* = 1484/2728) compared to the control group (51%, *n* = 71,826/139,835, *p* = 0.002).

**Table 1:** Incidence rates per each age years in for males and females

Age of epilepsy onset	Female (*n* = 69,253)		Male (*n* = 73,310)		Total (*n* = 142,563)	
	Number of initial diagnoses	Incidence rate per 100,000 (95% CI)	Number of initial diagnoses	Incidence rate per 100,000 (95% CI)	Number of initial diagnoses	Incidence rate per 100,000 (95% CI)
< 1	132	191 (161, 226)	158	216 (184, 252)	290	203 (181, 228)
1	231	334 (293, 379)	216	295 (258, 337)	447	314 (286, 344)
2	143	206 (175, 243)	184	251 (217, 290)	327	229 (206, 256)
3	109	157 (130, 190)	134	183 (154, 217)	243	170 (150, 193)
4	104	150 (124, 182)	131	179 (151, 212)	235	165 (145, 187)
5	107	155 (128, 187)	116	158 (132, 190)	223	156 (137, 178)
6	83	120 (97, 149)	100	136 (112, 166)	183	128 (111, 148)
7	84	121 (98, 150)	108	147 (122, 178)	192	135 (117, 155)
8	71	103 (81, 129)	89	121 (99, 149)	160	112 (96, 131)
9	100	144 (119, 176)	138	188 (159, 222)	238	167 (147, 190)
10	80	116 (93, 144)	110	150 (124, 181)	190	133 (116, 154)

### Prevalence and the risk of cognitive impairment in children with epilepsy

**Fig. 3:**
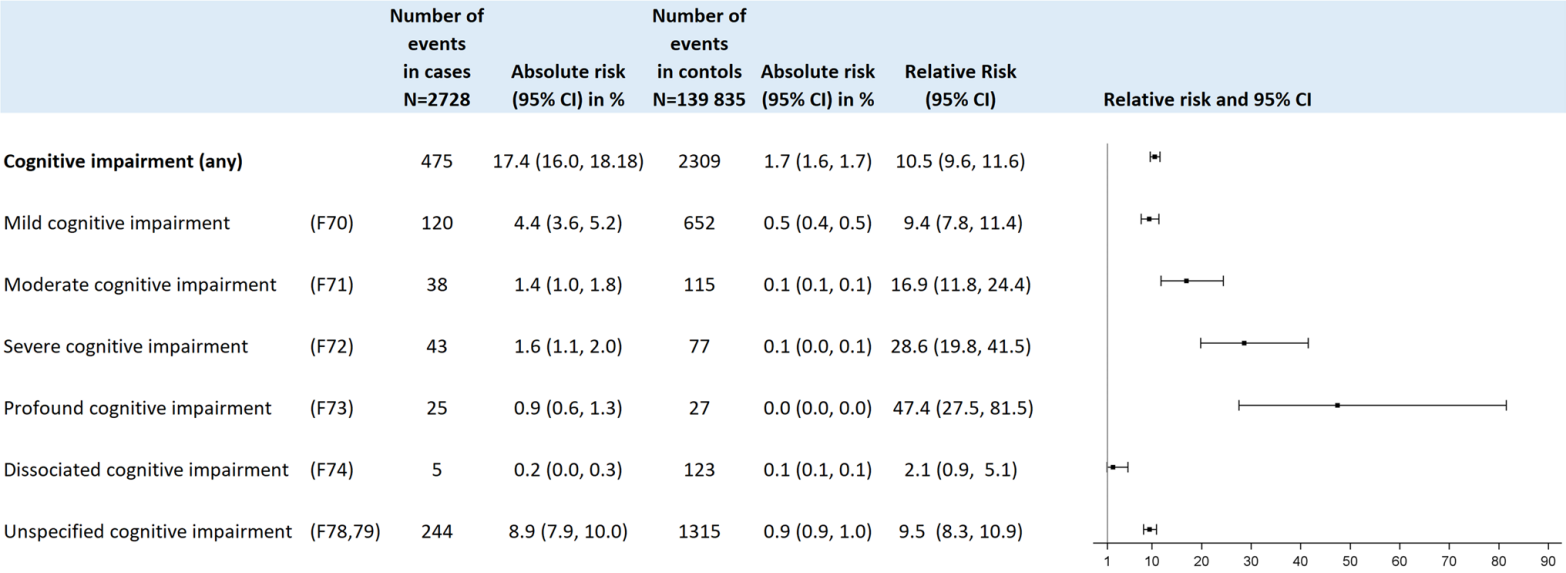
The risk of cognitive impairment in children with epilepsy compared to age-matched controls. The risk of cognitive impairment was increased by factor 10.5 in patients with epilepsy. Epilepsy patients revealed most likely not further specified (F78, 79) or mild (F70) cognitive impairment. The risk of profound cognitive impairment (F73) was almost increased by factor 50 compared to age-matched controls

Two thousand seven hundred and eighty-four (2.0%) children within the study cohort were identified as having cognitive impairment. Figure 3 summarizes the prevalence of different categories of cognitive impairment in 2728 cases with epilepsy and 139,835 control children and the relative risk for cognitive impairment. The prevalence of cognitive impairment was higher in children with epilepsy than in controls for all categories of severity (RR 10.5, 95% CI 9.6, 11.6). Overall, prevalence of cognitive impairment in the epilepsy group was 17.4% (475/2728), whereas it was 1.7% in the control group (2309/139,835). The incidence rate of cognitive impairment per each age year for both patients with epilepsy and controls is depicted in Table 2.

**Table 2:** Incidence rate of cognitive impairment per each age year (children with epilepsy and controls)

Age at onset of cognitive impairment	Cases						Controls					
	Female (*n* = 1244)		Male (*n* = 1484)		Total (*n* = 2728)		Female (*n* = 68,009)		Male (*n* = 71,826)		Total (*n* = 139,835)	
	Number of initial diagnoses	Incidence rate per 10,000 (95% CI)	Number of initial diagnoses	Incidence rate per 10,000 (95% CI)	Number of initial diagnoses	Incidence rate per 10,000 (95% CI)	Number of initial diagnoses	Incidence rate per 10,000 (95% CI)	Number of initial diagnoses	Incidence rate per 10,000 (95% CI)	Number of initial diagnoses	Incidence rate per 10,000 (95% CI)
< 1	4	322 (93, 856)	9	606 (300, 1168)	13	477 (270, 823)	34	50 (36, 70)	34	47 (34, 66)	68	49 (38, 62)
1	17	1367 (838, 2194)	25	1685 (1132, 2487)	42	1540 (1136, 2079)	55	81 (62, 105)	70	97 (77, 123)	125	89 (75, 107)
2	20	1608 (1028, 2486)	38	2561 (1862, 3504)	58	2126 (1644, 2743)	63	93 (72, 119)	69	96 (76, 122)	132	94 (80, 112)
3	21	1688 (1092, 2582)	19	1280 (808, 2004)	40	1466 (1074, 1995)	60	88 (68, 114)	94	131 (107, 160)	154	110 (94, 129)
4	24	1929 (1286, 2869)	28	1887 (1298, 2724)	52	1906 (1452, 2495)	94	138 (113, 169)	187	260 (226, 300)	281	201 (179, 226)
5	22	1768 (1156, 2678)	34	2291 (1634, 3194)	56	2053 (1580, 2660)	93	137 (112, 168)	210	292 (255, 335)	303	217 (194, 242)
6	16	1286 (776, 2096)	30	2022 (1409, 2882)	46	1686 (1262, 2246)	80	118 (94, 147)	166	231 (198, 269)	246	176 (155, 199)
7	28	2251 (1549, 3247)	31	2089 (1465, 2960)	59	2163 (1676, 2784)	73	107 (85, 135)	153	213 (182, 250)	226	162 (142, 184)
8	15	1206 (714, 1998)	27	1819 (1242, 2645)	42	1540 (1136, 2079)	92	135 (110, 166)	160	223 (191, 260)	252	180 (159, 204)
9	14	1125 (653, 1899)	22	1482 (969, 2247)	36	1320 (949, 1827)	92	135 (110, 166)	189	263 (228, 303)	281	201 (179, 226)
10	12	965 (533, 1698)	19	1280 (808, 2004)	31	1136 (796, 1614)	86	126 (102, 156)	155	216 (184, 253)	241	172 (152, 196)

The relative risk related to epilepsy increased by severity of cognitive impairment. While the relative risk for mild cognitive impairment in the epilepsy group was 9.4 [95% CI 7.8, 11.4], the RR for profound cognitive impairment was 47.4 [95% CI 27.5, 81.5] (Fig. 3). About half of the cognitive impairment diagnose was coded with ICD F78 or ICD F79 precluding information on the severity level. In the majority of patients with epilepsy, mild cognitive impairment was the most common grade of severity accounting for 120 out of 2728 (4.4%). More severe cases were encountered in 1.4% (moderate impairment), 1.6% (severe impairment) and 0.9% (severest impairment) of the cases. In 8.9% of the epilepsy cases, cognitive impairment was coded without documentation of the grade of severity (Fig. 3).

### The impact of age of epilepsy onset on the relative risk for cognitive impairment in epilepsy

**Fig. 4:**
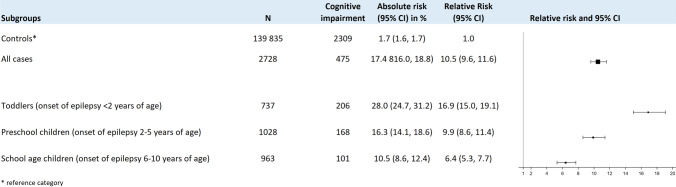
Risk of cognitive impairment in children with epilepsy compared to controls by age groups. There is a gradual increased absolute and relative risk for toddlers with epilepsy to have cognitive impairment compared to preschool—and school children

To assess the association of cognitive impairment and age of epilepsy onset, we stratified the analysis by age group (Fig. 4). The relative risk for cognitive impairment was highest in epilepsy cases with seizure manifestation within the first 2 years of life than in patients with seizure manifestation in preschool (2–5 years) and school (6–10 years) age (RR 16.9 vs. 9.9 and 6.4, respectively).

### The temporal relation of epilepsy onset and cognitive impairment

**Fig. 5:**
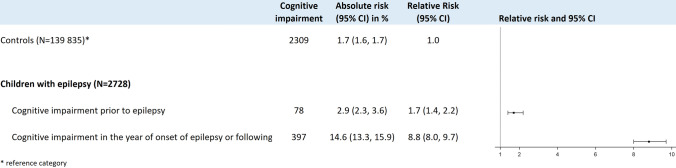
Risk of cognitive impairment before and after epilepsy diagnosis compared to the general risk of cognitive impairment. The majority of patients received the diagnosis of cognitive impairment after the diagnosis of epilepsy

Considering the temporal sequence of the diagnoses of epilepsy and cognitive impairment, the prevalence of cognitive impairment before epilepsy diagnosis was slightly higher (2.5%, 78/2728) compared to the prevalence of the general population (1.7%, 2309/139,835) with an increased risk of RR 1.7 (95% CI 1.4, 2.2). The risk for cognitive impairment in the years at and after the epilepsy diagnosis was significantly increased (RR 8.8 (95% CI 8.0, 9.7)) compared to cognitive impairment in children without epilepsy (Fig. 5).

## Discussion

In the present study, we analyzed a birth cohort of considerable size based on healthcare data. We observed a cumulative incidence of epilepsy up the age of 10 slightly above the upper range of other studies with different approaches for assessment. We found an about ten-fold higher risk for cognitive impairment in children with epilepsy than in children without epilepsy, which was most pronounced for severe cognitive impairment. The risk for cognitive impairment in epilepsy cases was higher in younger children. Though the risk for cognitive impairment was slightly increased prior to the onset of epilepsy compared to controls, cognitive impairment mainly appears to follow the onset of epilepsy.

### Epidemiology of epilepsy and cognitive impairment

Reported incidences for epilepsy in children vary from 41/100,000 to 187/100,000 [23]. Our incidence estimates of 191 [95% CI 185, 200] per 100,000 person years in the first 10 years is higher than the previously reported. There are several possible reasons for this: different age ranges [24], different case definitions since some require the use of ASM [25], or potential misclassification, since no individual case validation was possible in our data. The latter would account for an overestimation for prevalence and an underestimation of the risk for cognitive impairment. In accordance with others, we observed the highest risk for onset of epilepsy in the first year of life [26, 27]. Furthermore, higher incidence and prevalence rates for epilepsy are more likely to occur in claims data analyses compared to field data research. This was recently shown in a large claims-based analysis in adults with epilepsy providing different estimates depending on diagnosis criteria [28]. Another explanation for the high incidence rate of epilepsy in the present study might be related to the search strategy which might appear to “loose”. One could argue i.e. to include only G40 patients who receive ASM. However, this could led to false exclusion of patients with mild epilepsy phenotypes as rolandic epilepsy, who might be left untreated in some circumstances [29]. Nevertheless, a selection bias toward false classified patients as epileptic patients cannot be excluded in the present study. Approximately 3% of children of school age are suspected to have cognitive impairment [30], which is slightly higher than our estimate with 2%. Since diagnosis of cognitive impairment requires intelligence testing, the physicians’ diagnostic coding is likely to be valid. Some non-differential under-ascertainment appears possible because of failure to send all cases with developmental delay for IQ testing.

### The prevalence and risk of cognitive impairment in epilepsy

Population-based studies in cohorts of children with epilepsy found cognitive impairment or subnormal cognitive function in about one third of the cases [6, 11, 12]. The prevalence of cognitive impairment in children with epilepsy in absence of a control group does not indicate the risk. Assessment of the risk requires a control group of children without epilepsy. Identical ascertainment for cognitive impairment for both the epilepsy cohort and the non-epilepsy cohort is mandatory for valid risk estimates. This is a unique feature in our data. We could not identify previous population-based studies including a contemporary control group in the same health insurance setting. Although only 17.4% of our cases had cognitive impairment, their risk for cognitive impairment compared to children without epilepsy was increased by a factor of about ten. A recent claim-based analysis on developmental speech and language disorders revealed a high prevalence in epileptic patients’ age 0–19 years [31]. Given the close association between language development and cognition, these results supports or our findings.

### Age-dependent risk of cognitive impairment in children with epilepsy

Cognitive impairment is more common in children with early epilepsy onset, and in children with high burden epilepsy for example in children, who develop drug-resistant seizures [32–34]. In early onset cases, the following factors might impact cognitive function: etiology, seizure frequency, presences and absence of EEG-status and the type and total load of ASM. The latter is supposed to be high in this age group according to high incidence of medically refractory cases due to above mentioned etiologies [35]. The design of the present study does not allow to further dissect the role of these different parameters. Data on genetic epilepsies i.e. Dravet syndrome suggest that epilepsy-specific parameters like seizure frequency contribute less to cognitive decline than the underlying biological cause (= etiology) itself [36].

### The temporal relation of cognitive impairment and onset of epilepsy

The risk of cognitive impairment was slightly higher prior to the diagnosis of epilepsy than in children without epilepsy, but further increased during the course of disease suggesting that epilepsy-specific parameters as seizure frequency may increase the risk of cognitive impairment. Nevertheless, this association might also be due to increased awareness to intellectual impairment triggering more systematic cognitive testing once the diagnosis of epilepsy is established. Furthermore, the diagnosis of intellectual impairment by means of an ICD F7* diagnosis cannot be established within the first years of life and these patients might diagnosed instead with the ICD code of global developmental delay (R62.8), which was not considered in our study. Patients with an increased risk of cognitive impairment before seizure onset suggest strong contributing factors on cognitive impairment besides typical epilepsy-related factors (as seizure frequency and drug treatment). Most likely, etiological factors causing cognitive impairment are also responsible for the predisposition of epileptic seizures. For example, patients with structural brain damage, i.e. due to perinatal hypoxemia or malformations of cortical development may reflect cognitive impairment before seizure initiation. For patients with epilepsies associated with mainly monogenetic causes, it was even proposed to call these syndromes ‘Developmental brain disorders with the comorbidity of epilepsy’ to pronounce the high impact of other underlying dysfunction beside epilepsy and to acknowledge the importance of the neurobiological basis in causing both seizures and cognitive impairment [8]. Nevertheless, cognition might deteriorate upon seizure onset in some circumstances. This bidirectional relationship [37] between epilepsy and cognition is especially visible in patients with structural brain damage and new onset West-syndrome: while developmental delay is usually present prior seizure onset, the beginning of epileptic spams and the EEG patterns of hypsarrhythmia coincides with further cognitive deterioration in the patients.

### Homogeneity of the analyzed cohort

Ninety percent of the German population is insured by statutory health insurances. There are no obligations with respect to socio-economic status to join either of the statutory insurances suggesting homogeneity of the insured population. Eventually, patients insured by private insurances (10%) have higher income compared to people in statutory insurances. Incidence rates of neurological conditions do not differ between these two types of insurances. Furthermore, there are no differences in the proportion of insured children and adolescents and the amount of utilization of the health care system [38]. External validity is further supported by Hoffman et al., who compared adults with different statutory health insurance in Germany. They did not find a difference regarding educational level, but a slightly higher prevalence of chronic diseases in BARMER insured persons [39].

### Case definition

In the present study, we did not use G41 (status epilepticus) as a diagnosis of epilepsy unless G40 was concomitantly coded as described in the method section. Status epilepticus in children is most often caused by acute conditions (most commonly by fever followed by CNS infections, trauma, etc.) [40]. In the case of recurrent status epilepticus in children, an underlying epilepsy is evident in the vast majority of cases [41] and these patients are most likely covered by the G40 case definition we provided within the method section. Accordingly, we considered status epilepticus as a diagnosis of a prolonged (mostly symptomatic) seizure rather than a chronic disease as epilepsy. It could be further argued for excluding patients with G41 and without G40 diagnosis from the control group as they might carry an increased risk of later neurodevelopmental impairment [42]. Consequently, one would have to consider excluding other neurological conditions, too. The term “control” group might be misleading in this context: “control group” means that this group should not contain patients with epilepsy but does not imply the inclusion of solely healthy subjects. The true increase in cognitive impairment by epilepsy might thus be rather underestimated. Febrile convulsions (R56.1) were included in the control group unless the diagnosis of epilepsy (G40) was coded as the majority of cases with febrile convulsions are not associated with epilepsy and have a favorable neurodevelopmental outcome [43].

### Strengths and limitations

There are few population-based data on the incidence of epilepsy and diagnoses of cognitive impairment in pre-pubertal children, even fewer with a complete follow-up throughout the first 10 years of life and inclusion of control subjects. Such data allow for valid assessment of the population attributable risk for epilepsy, the temporal sequence of comorbidities and comparison to the risk in children without epilepsy.

Limitation of the analysis is the structure of the dataset, which precludes individual case validation for the diagnosis of epilepsy as well as for the diagnosis of cognitive impairment. Misclassification, therefore, appears possible. Incorrect ICD coding of patients with seizures not fulfilling the criterion of chronic epilepsy as defined by the ILAE might be taken in account [44]. However, the accuracy of identification of epilepsy cases using healthcare administration data based on ICD G40* yielded a positive/negative predictive value, sensitivity and specificity > 80% in the majority of studies [20, 45, 46].

Regarding cognitive impairment, we have no data on whether IQ testing was performed equally often in children with and without epilepsy. The prevalence of cognitive impairment within entire study population studied, however, was comparable with the literature. Although, BARMER is a major statutory health insurer in Germany limitations regarding external validity cannot be definitively ruled out.

The definition of the pre-pubertal period might be misleading in patients with epilepsy. Especially in patients with epilepsy and additional cognitive impairment, puberty might precocious physiologically expected [47].

We did not dissect the epilepsy to further syndromes and etiologies, which is due to the nature of the ICD classification system. Epilepsy classification according to the ILAE has changed significantly over time and although ICD classification divides epilepsy into focal and generalized syndromes, this appears to be only a rough approach of categorizing epilepsy syndromes [48–50]. Furthermore, epidemiologic studies as performed here cannot prove causality, and even less a role of specific underlying mechanism. Thus, we describe associations and present possibly explanations rather than proving causality or even certain biological mechanisms.

## Conclusions

The strength of the association of epilepsy and cognitive impairment appears to be a factor of about ten. Children with early epilepsy manifestation are especially at high risk for the diagnosis of cognitive impairment. The diagnosis of cognitive impairment was coded after epilepsy diagnosis in the majority of the cases. This might reflect different explanations as (1) an increased awareness of cognitive comorbidities once the epilepsy diagnosis is established, (2) the difficulty in diagnosing the F7* code in very young children, and (3) the impact of epilepsy-specific parameters in cognition itself in some cases or a common cause for both (4).

## Data Availability

This is an analysis on healthcare data, not owned by the authors. BARMER gives remote access to their data to researchers who provide a methodologically sound proposal for use in achieving the goals of the approved proposal.

## Abbreviations

ICD: International classification of diseases
IQ: Intelligence quotient
ATC: Anatomical therapeutic chemical classification
ASM: Antiseizure medication
CI: Confidence interval
RR: Relative risk
SD: Standard deviation
